# Understanding Artificial Intelligence (AI) for the Electrophysiologist

**DOI:** 10.1016/j.ipej.2026.01.010

**Published:** 2026-01-30

**Authors:** Charulatha Ramanathan, Natalia A. Trayanova

**Affiliations:** a Carelog, 2945 Mokelumne Ct, Manteca, CA, 95337, USA; b Biomedical Engineering, Alliance for Cardiovascular Diagnostic and Treatment Innovation (ADVANCE), Johns Hopkins University, Baltimore, MD, USA

## Abstract

Artificial intelligence (AI) is increasingly incorporated into clinical electrophysiology, Applications now span automated ECG interpretation, arrhythmia detection, risk stratification, procedural planning, and workflow support. At the same time, variability in methodological rigor, validation standards, and clinical integration has led to uncertainty regarding how these tools should be interpreted and used in clinical practice.

This review provides a practical primer on AI for electrophysiologists, with the goal of supporting informed evaluation and responsible clinical adoption. We outline the historical evolution of AI, from rule-based systems to contemporary machine learning, deep learning, and emerging generative AI and large language models. Core methodological concepts are reviewed, with emphasis on data provenance, labeling, validation strategy, and the distinctions between analytical performance and clinical utility. Common failure modes are examined, including bias and lack of representativeness, overfitting, limited interpretability, workflow misalignment, and overstatement of clinical readiness.

We further discuss how regulatory agencies evaluate AI-based electrophysiology tools, what regulatory clearance establishes, and what it does not. Particular attention is given to the implications of static model review, device-specific validation, and intended use constraints, and to the continuing responsibility of clinicians in appropriate deployment and oversight.

Finally, we consider future directions for AI in electrophysiology, including individualized modeling approaches, expert decision support in resource-constrained settings, and applications aimed at improving efficiency and access to care. This review provides electrophysiologists with a practical framework to interpret current AI evidence and to actively guide how AI is evaluated, adopted, and translated to clinical practice.

## Highlights

• AI performance in EP is determined by model architecture, data provenance, label quality, and validation strategy.
• Most EP AI failures stem from biased or narrow datasets, overfitted models, and misaligned clinical workflows.
• FDA clearance confirms analytical performance under defined conditions, but not clinical effectiveness or generalizability.
• Electrophysiologists must evaluate AI tools by examining intended use, data–hardware match, failure modes, and clinical risk.
• Future value in EP AI lies in patient-specific modeling, and extending expert-level support to low-resource settings.

## 1 Introduction

Artificial intelligence (AI) is transforming clinical medicine at an unprecedented pace. The convergence of large-scale digitized health records and advanced computational power now enables AI to detect clinically relevant patterns in physiologic signals that would be impossible for humans to discern manually. The momentum is striking: while the FDA granted its first AI algorithm clearance in 1995, more than 1200 have been approved to date, with approximately 30 new algorithms now entering the market each month.

AI applications in electrophysiology (EP) span the entire care pathway including early detection of arrhythmias through FDA-cleared wearable devices and point-of-care tools for atrial fibrillation screening [1]; risk stratification for sudden cardiac death [2], screening for contractile dysfunction [3]; procedural planning outcome prediction [4] and real-time intraprocedural guidance during catheter ablation [5]. Emerging applications include patient-specific digital twinning for personalized arrhythmia treatment strategies [6,7]; multimodal AI integrating electrocardiographic, imaging, and clinical data for personalized risk assessment [8] near-term prediction of life-threatening ventricular arrhythmias from ambulatory monitoring [9] and new explainable AI models that provide mechanistic insights into arrhythmia substrates.

This article provides an overview of AI for electrophysiologists from a perspective of two overlapping audiences - those involved in the development/evaluation of AI enabled clinical tools, and those who will increasingly encounter and use these tools in everyday clinical practice. By grounding core AI concepts in familiar EP signals and workflows, this review aims to equip electrophysiologists with a practical framework to interpret AI-based studies, understand common sources of error and bias, and appropriately interpret regulatory clearances and their implications for clinical practice. Subsequent articles in this series will examine disease specific and setting specific applications of AI across EP, including atrial fibrillation, ventricular arrhythmias and sudden cardiac death, electrophysiology laboratory workflows, implantable devices and remote monitoring, and ethical and explainability considerations.

## 2 An AI primer for Electrophysiologists

AI is a broad term used to describe computational systems that perform tasks such as pattern recognition, classification, prediction, or synthesis in ways that are not explicitly hard coded, and typically beyond manual cognition or processing. Not all computational tools used in medicine are AI. Traditional data analytics, descriptive statistics, and rule-based algorithms apply predefined logic and calculations but do not learn from data, and are not considered AI.

To make sense of the growing number of AI based tools in EP and their implications for clinical practice, it is useful to step back and examine how AI has evolved, which problems it has been able to solve reliably, and where its limitations have persisted. This section provides a concise primer on the historical development of AI, the major methodological approaches that underpin contemporary clinical tools, common pitfalls and what to keep in mind as an EP either developing or using AI in their practice.

### 2.1 Brief history of AI for EP

One of the earliest practical applications of computer-based intelligence was automated ECG interpretation, using rule-based waveform morphology and interval measurements [10]. These early systems demonstrated that clinically meaningful signal features could be extracted reproducibly at scale when acquisition was standardized and interpretive logic was well understood, establishing the template.

In the 1970s and 1980s, automated ECG analysis expanded from measurement to diagnostic inference through expert systems that encoded rhythm classification and diagnostic logic using symbolic rules [11]. Although this is not classical AI, it formed the foundation of automation for clinicians.

During the late 1980s and 1990s, increased computational capacity enabled a shift toward statistical pattern recognition in ECG analysis [12]. Feature-based classifiers and early neural networks were applied to ECG interpretation; feature-based models relied on clinician-defined measurements, whereas early neural networks drew inspiration from the brain, using many simple “neuron-like” mathematical units connected in layers. Each unit performs a small calculation on the ECG signal, and by adjusting the strength of the connections between units—analogous to synaptic weighting—the network learns to recognize more complex patterns from training examples. Therefore, instead of relying on fixed diagnostic rules defined *a priori* by domain experts, these models used predefined ECG features, such as waveform morphology and interval measurements, to learn how different combinations of features corresponded to rhythms or diagnoses based on prior examples [13]. This transition, from expert-defined logic to data-driven learning from examples, formed the basis of what is now referred to as machine learning (ML).

At this stage, nearly all work focused on the surface ECG rather than intracardiac signals. Surface ECGs were far more abundant, standardized, inexpensive to acquire, and annotated at scale, enabling the large datasets required for algorithm development. In contrast, intracardiac electrograms were limited to procedural environments, lacked public repositories, and had considerably more variation in catheters, mapping systems, and acquisition settings, making them harder to use for early AI research. As a result, the surface ECG became the dominant substrate for AI development in electrophysiology.

In the 2000s and 2010s, the digitization of large ECG repositories [14,15] and advances in computational infrastructure enabled models to operate directly on raw or minimally processed signals. Deep learning approaches, a class of neural networks with multiple hierarchical layers, reduced reliance on clinician-selected features and instead learned signal representations directly from data [16]. A growing number of ECG-based algorithms in clinical use are built on deep learning frameworks and supported by formal validation evidence [17]. It must be noted that the features learned by these models are often *not* visually apparent from the standard ECG, which may reasonably prompt clinician skepticism.

Today, the scope of AI in medicine has expanded beyond signal interpretation to include generative AI applications that support clinical workflows and decision making. Large language models operate primarily on text rather than physiologic signals and are trained on massive corpora using self-supervised learning to model relationships between sequences of words or tokens. Although they do not understand physiology, their ability to summarize information, draft reports, and synthesize clinical text has created new categories of workflow support that complement—rather than replace—signal-based AI [18,19]. These applications differ fundamentally from prior signal-focused models in that they operate primarily on text and multimodal clinical data rather than physiologic signals, and their clinical value is tied to accuracy, reliability, and appropriate human oversight rather than direct pattern recognition in waveforms [18].

### 2.2 Deep dive into AI methodologies

#### 2.2.1 Data

Any discussion of AI methods must begin with data, because data define the types of approaches that can work, the framework for training, and how reliably insights translate to clinical practice. Relevant, sufficient, and well-characterized datasets are a prerequisite for AI algorithm development and strongly influence whether an AI application performs reliably in clinical settings. Experience from decades of automated ECG analysis has demonstrated that algorithmic performance reflects not only underlying electrophysiologic phenomena, but also how data are acquired, curated, annotated, and evaluated [15,20].

***Training data and test data: learning versus evaluation:*** AI models learn from training data, which consist of paired input data and reference labels (ground truth) that define the task the algorithm is expected to learn. The input may be a surface ECG or intracardiac electrogram, and the reference label may be a rhythm diagnosis, ECG interpretation, or clinical outcome associated with that signal. Note that in many EP applications, the ground truth represents an adjudicated or surrogate reference rather than a definitive physiologic truth. Rhythm labels, ECG interpretations, and even procedural endpoints are frequently derived from expert review, consensus panels, or downstream clinical decisions, each of which introduces uncertainty and potential bias [21]. In such settings, AI models are trained to reproduce the reference standard as defined, not necessarily the underlying mechanism. Recognizing when labels reflect expert consensus or surrogate outcomes rather than objective truth is essential for interpreting model performance and clinical applicability.

During training, the model is repeatedly exposed to these signal–truth pairs and adjusts its internal parameters to reduce disagreement between its predictions and the provided labels. Over time, the model learns which signal characteristics are consistently associated with specific labels in the training data. This process is conceptually analogous to how human electrophysiologists learn ECG interpretation during training. Reviewing tracings in isolation provides little value; learning occurs by reviewing tracings alongside diagnoses, clinical context, and outcomes.

The implications of this process are critical. If labels are inconsistent, ambiguous, or reflect institutional practice rather than underlying electrophysiology, the model will learn those patterns or get confused. Likewise, if certain rhythms, patient populations, or acquisition conditions are underrepresented in the training data, the model's performance will be biased toward the patterns it has seen most frequently. These limitations are properties of the training data used to define the task rather than of the algorithm itself [22,23].

To evaluate whether learning has generalized beyond memorization of the training data, model performance must be assessed on separate test datasets that were not used during training. Test data are constructed in the same signal–label format but represent previously unseen examples. Performance on such datasets provides an estimate of how the model is likely to behave when applied to new patients in clinical practice [22,23]. Training and test datasets must not overlap, and each frozen version of a model should be evaluated on an independent or external test dataset to ensure unbiased assessment of performance.

***Data curation and annotation: why raw data are not enough:*** Raw physiological signals data of any kind - ECG, intracardiac electrograms, imaging, or EHR-derived clinical variables are rarely suitable for AI development without careful curation. Data curation involves selecting representative examples, excluding corrupted or noninformative inputs, and standardizing formats and metadata. Annotation assigns clinically meaningful labels, often through expert review or adjudication. Experience with widely used ECG databases has shown that labeling errors and ambiguities are common, even in benchmark datasets, and can propagate into downstream analyses if not addressed explicitly [20,21]. The same issues arise across other modalities. Imaging poses additional challenges: segmentation-dependent labels vary by operator and institution, and in some applications, using raw imaging intensities rather than heavily processed or segmented images may reduce annotation bias. At the same time, training exclusively on “clean,” narrowly curated datasets can reduce generalizability, as models fail when exposed to real-world variability. These limitations underscore a broader principle in AI for EP: regardless of the modality, data quality and data representativeness jointly determine whether an algorithm will behave reliably in clinical practice.

***Structured and unstructured data:*** AI applications in EP may use structured data, such as waveform measurements, device parameters, and coded diagnoses, or unstructured data, such as clinical notes and procedure reports. Structured data are easier to analyze and validate but may omit important clinical context, whereas unstructured data contain richer information at the cost of variability and ambiguity. Signal-based AI models primarily operate on structured physiologic data, whereas workflow-oriented and documentation-focused AI systems rely largely on unstructured text. These data types require different modeling approaches and validation standards and should not be conflated when interpreting AI claims [24].

#### 2.2.2 AI models

AI applications in electrophysiology draw on a limited number of underlying computational model classes. These models differ in how they represent information, how they learn from data, and what types of clinical problems they are best suited to address. Understanding these distinctions is essential for interpreting AI-based studies and for evaluating whether a given approach is appropriate for a specific EP application.

***Rule Based Systems:*** Rule based systems, historically referred to as expert systems, are a foundational class of AI methods grounded in explicit logic and interpretability [25]. Decision logic is specified *a priori* by domain experts and does not change with additional data. For identical inputs, these systems produce identical outputs. An example of a prevalent rule-based system, automated rhythm discrimination in implantable cardioverter defibrillators relies on hierarchical rule based logic incorporating specific and known criteria [26]. Rule based systems perform reliably when diagnostic criteria are well defined and consensus driven. Their limitations emerge in edge cases not anticipated by the rule set, contributing to inappropriate therapy in scenarios such as atrial fibrillation with rapid ventricular response or polymorphic ventricular tachycardia.

***Traditional Machine Learning (ML) Models:*** These models learn statistical relationships between predefined clinical variables and outcomes using labeled datasets. Unlike rule based systems, the decision logic is not explicitly programmed, but is learned from data. Importantly, the inputs to these models are still variables selected and defined by subject matter experts (e.g., ECG measurements). Common approaches used in electrophysiology include logistic regression, decision trees, random forests, and gradient boosting methods.

In practice, ML applications in EP fall into three broad learning paradigms. Supervised learning trains models on labeled examples to classify rhythms or predict outcomes. Unsupervised learning is used more selectively to identify latent phenotypes or patient subgroups without predefined labels. Unsupervised models may find relevant patterns/relationships that otherwise remain undiscovered.

Reinforcement learning, which optimizes decisions through trial and feedback, remains largely investigational in this domain. These models can flexibly weight variables, capture nonlinear relationships, and model interactions that are difficult to analyze manually or with traditional mathematical approaches. They are well suited to problems involving structured data and moderate dataset sizes, offering a balance between performance and interpretability, but are limited in their ability to capture complex signal-level patterns without extensive feature engineering.

An example of a supervised ML model (random forest, logistic regression) trained on structured clinical registry data (demographics, comorbidities, medications, and lab values) have been used to improve stroke risk prediction in atrial fibrillation patients [27].

***Deep Learning (DL) Models:*** These are a subclass of ML methods based on neural network architectures, in which signals are processed through multiple stacked computational layers that progressively transform the input data. Through this process, models learn hierarchical signal representations, ranging from local voltage and timing relationships to higher-order patterns that reflect more complex physiologic signatures. Unlike traditional ML models, where input variables are predefined and fixed, deep learning models learn both what features matter and how those features relate to clinical outcomes directly from the input data during training [28].

A direct consequence of this representation learning is that the features driving model predictions are often not directly verifiable through visible ECG morphology or conventional measurements. Whereas rule based systems and traditional ML models allow decision logic to be traced to specific intervals, amplitudes, or thresholds, deep learning models integrate information across time, leads, and signal scales in ways that do not map cleanly to human-interpretable features. As a result, clinically relevant patterns identified by these models may not correspond to recognizable waveform changes, even when they are reproducible and statistically robust within the data. This disconnect between model inference and visual interpretability represents a fundamental shift in how evidence is derived from physiologic signals and underscores the need for careful validation and contextual clinical judgment.

Deep learning models perform best when trained and evaluated on large, diverse, and well-curated datasets; as with any data-driven method, “garbage in, garbage out” applies. Deployment conditions must also resemble the environment in which the model was developed to maintain performance. A DL model's biggest limitation is limited interpretability in conventional electrophysiologic terms, and model behavior may be difficult to anticipate under distributional shift, such as changes in acquisition hardware, noise characteristics, or patient populations. These limitations place greater emphasis on rigorous external validation, assessment of generalizability, and clearly defined clinical use boundaries compared with rule based or traditional ML approaches.

Deep learning has been incorporated into commercially deployed ECG analysis systems for arrhythmia detection and classification. Several FDA-cleared ambulatory and mobile ECG platforms use DL models trained on large, annotated ECG datasets to identify atrial fibrillation or low ejection fraction from ECG recordings, and are now widely used in ambulatory monitoring and remote care workflows [1,16,29].

**Fig. 1:**
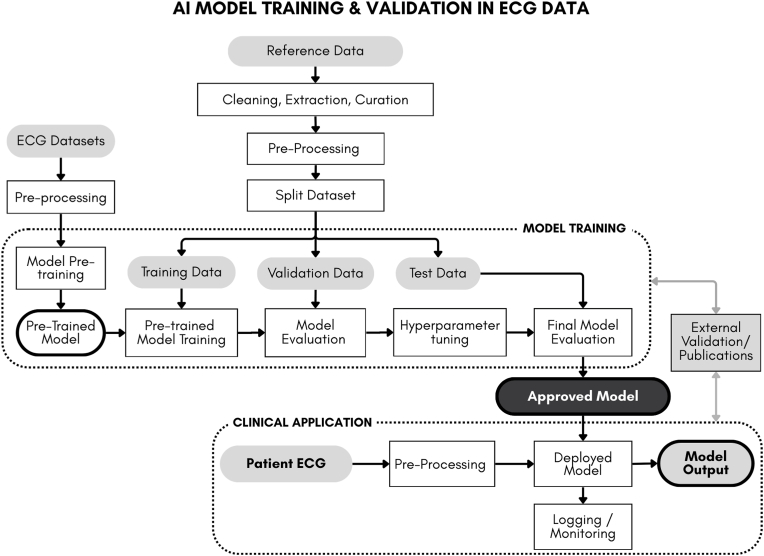
**Illustrated Workflow: Example of AI model training/validation in ECG data.** Model development typically begins with assembling large ECG datasets for model pretraining, allowing the AI model to learn general ECG structure and signal patterns before task-specific training. For the clinical task, labeled reference datasets relevant to the specific task are created(e.g ECG–echo pairs for low ejection fraction algorithms or ECG–clinical outcome pairs for arrhythmia classification). This reference set is the ‘truth data’ that the model will be further trained and validated on. After cleaning and preprocessing, the reference data are split into training, validation, and test sets, commonly using a 70/20/10 proportion. The model is trained on the training set, tuned on the validation set, and evaluated on the test set to estimate performance for the frozen model. Once validated, the finalized model is prepared for regulatory submission or publication. During clinical deployment, new ECGs follow the same preprocessing pipeline and are analyzed by the deployed model, with ongoing monitoring to detect performance drift or facilitate further model improvements needed.

Fig. 1 shows an illustrative workflow (generalized) of an AI DL model training for ECG.

**Generative AI (Gen AI) and Large Language Models (LLMs):** These models represent a distinct class of AI systems designed to generate new content rather than to classify or predict predefined clinical outcomes. LLMs are the most prominent example and are trained using self supervised learning on massive corpora of unstructured text to model statistical relationships between sequences of words or tokens [30,31]. Unlike traditional ML or DL models, LLMs do not learn from labeled clinical inputs or endpoints. Instead, they learn to predict the most likely next token (output) given prior context, prioritizing contextually appropriate language without explicit grounding in underlying biologic or causal mechanisms [25]. As a result, while LLMs may generate responses that resemble expert clinical reasoning, these responses are not derived from physiologic modeling and cannot be assumed to reflect mechanistic understanding [32,33]. This limitation underlies the phenomenon of hallucination [34], in which generated outputs are syntactically coherent but factually incorrect or unsupported by evidence. Therefore, Gen AI systems currently are best understood as workflow and communication tools rather than diagnostic or predictive models. Emerging applications include clinical documentation, summarization of medical records, guideline navigation, research assistance, and patient communication support [19,35].

#### 2.2.3 Fits, starts and failures: common pitfalls in clinical AI

Understanding why prior AI approaches failed to translate into routine EP practice is essential for evaluating/adopting AI into clinical practice. Across multiple generations of AI tools, failures have emerged at three distinct but interrelated levels: data, model, and clinical translation. These recurring pitfalls, including bias, overfitting, limited interpretability, and workflow misalignment, continue to shape whether AI delivers sustained clinical value.

***Bias and lack of representativeness in the data:*** Many early AI models were trained on narrowly curated datasets that did not reflect real world patient diversity, device heterogeneity, or acquisition environments. Models developed using large but single health system datasets often demonstrated strong internal performance, yet failed when applied to ECGs acquired from different devices, lead configurations, or patient populations. This exposed reliance on dataset specific artifacts rather than robust electrophysiologic patterns and highlighted bias and limited representativeness as foundational barriers to generalizability [15,20].

Mitigation requires deliberate dataset design and validation strategy. Training datasets should incorporate diversity across institutions, devices, acquisition conditions, and patient populations. Performance should be evaluated across predefined subgroups and scenarios. External validation using data from independent health systems is essential before clinical deployment, and device claims should be limited explicitly to the populations and environments represented in the training data.

***Overfitting and misleading model performance:*** Overfitting has been a persistent limitation in many EP AI models. Overfitting occurs when an AI model captures patterns specific to the training dataset, such as device characteristics, signal preprocessing choices, labeling conventions, or institutional practice patterns, rather than generalizable EP phenomena. As a result, performance appears strong during development or internal validation but degrades when the tool is applied to ECGs acquired with different hardware, lead configurations, patient populations, or clinical workflows. Many models achieved high reported accuracy, sensitivity, or AUC during retrospective development and internal validation, yet showed substantial performance degradation during external testing or prospective evaluation [36]. Systematic evaluations of medical AI have shown that internally validated performance metrics frequently overestimate clinical readiness, contributing to premature translation and subsequent loss of clinician confidence [23,37,38].

Reducing overfitting requires both technical and study design safeguards. Models should be evaluated on truly independent datasets that differ meaningfully from the training data, including differences in site, device, and time period. Prospective validation, even at small scale, provides more informative evidence than repeated retrospective optimization. Reporting should include failure modes and subgroup performance, not only peak metrics, to prevent premature translation.

***Limited model interpretability and clinical plausibility*** Many AI models, particularly DL–based systems, generate outputs that are not directly verifiable using conventional markers such as visible ECG morphology, intervals, or amplitudes. A well known example is deep learning–based ECG algorithms that identify reduced left ventricular ejection fraction despite the absence of recognizable morphologic correlates on surface ECG, creating uncertainty around physiologic plausibility and mechanism [3]. This lack of interpretability becomes especially problematic when algorithmic outputs conflict with clinician judgment or are proposed for use in higher risk clinical decisions. In addition, the absence of clearly defined failure modes makes it difficult to anticipate when a model may behave unpredictably, reinforcing cautious or selective adoption in clinical practice [39].

Mitigation requires aligning model design and deployment with clinical risk rather than pursuing interpretability as an abstract goal. Application design should incorporate verification and oversight mechanisms such as interpretable intermediate outputs, human in the loop review, or co-pilot configurations in which AI augments rather than replaces clinician decision making [18,40]. Clear articulation of what a model does and does not measure, combined with post hoc analyses that assess physiologic plausibility and performance boundaries, can improve clinician trust without overstating mechanistic understanding or causal inference [41].

**Fig. 2:**
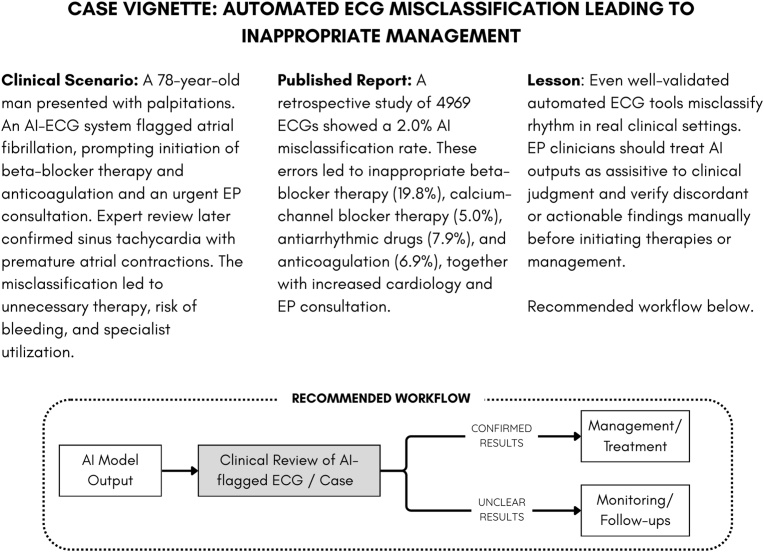
**Case vignette of ECG-AI misclassification and its clinical implications.** This figure illustrates how an automated AF-detection output led to inappropriate management before expert review. It incorporates evidence from a published study [42] quantifying the prevalence and consequences of such errors, highlights key lessons for electrophysiologists, and presents a conceptual “clinician-in-the-loop” workflow that positions AI tools as assistive rather than authoritative.

Fig. 2 presents a Case Vignette showing how an AI-generated AF label can misdirect management decisions, with a corresponding “clinician-in-the-loop” workflow illustrating the appropriate way to integrate such tools. Similar patterns of misclassification have been documented in practice [42].

***Application level failures: workflow and responsibility misalignment:*** Many AI tools failed because they disrupted established clinical workflows or did not address barriers in practice. There are many examples of such failure modes: devices that require parallel interpretation, cumbersome additional steps, or increased cognitive and operational burden without clear efficiency gains. In time sensitive diagnostic and procedural settings, even modest workflow friction limited sustained adoption. In addition, ambiguity regarding clinical responsibility when algorithmic outputs conflicted with physician judgment further constrained routine use, particularly for tools positioned as decision support [18].

Successful AI device translation requires not just strong engineering, but engaging the electrophysiologist and their support teams as early as possible. Validation must include not only algorithm performance but also protocols to translate into clinical practice, existing workflows and decision pathways, with clearly defined roles for the clinicians involved. AI tools should reduce, not increase, interpretive burden and should align with how electrophysiologists already make decisions. Explicit guidance on responsibility and escalation when outputs disagree with clinical judgment is essential for adoption [43]. When AI technologies are deployed across different practice or country contexts, additional evaluation of clinical workflows and local practice patterns is required. Without this, tools validated in one setting may fail to translate into routine use in another, regardless of algorithm performance.

***The ‘hype’ failure:*** In recent years, overstated claims of clinical readiness have stemmed from importing consumer-technology norms into healthcare, amplified by media cycles and rapid dissemination channels. The “fake it till you make it” culture may work in consumer domains, but in medicine it erodes clinician trust and risks patient safety. Several high-profile evaluations of medical AI have shown large drops in performance when internally developed models were applied prospectively or in external settings, underscoring the gap between promising prototypes and deployable clinical tools [22,23]. Regulatory bodies such as the FDA provide essential safeguards, but they cannot shoulder the entire burden.

Claims of readiness must match the strength of validation. Clear distinction between proof-of-concept, clinical validation, and real-world deployment is essential. Regulatory review, post-market surveillance, and ongoing performance monitoring remain necessary complements to model development and are critical to sustaining safe clinical use.

## 3 Clinical translation of AI tools

The clinical value of an AI system depends not only on methodological rigor, but on its successful translation into real practice. Translation spans the path from algorithm development to regulatory review and, ultimately, to everyday use where these tools influence diagnostic pathways, procedural decisions, and patient management. Understanding what regulatory clearance establishes, and what it does not, is essential for electrophysiologists who will be the end-users of these technologies.

### 3.1 What electrophysiologists need to know about AI regulatory clearance

Most AI-based electrophysiology tools cleared by the FDA (and applicable for other regulatory bodies) rely primarily on *retrospective analytical validation*, where algorithm outputs are compared to an established reference standard using metrics such as sensitivity, specificity, PPV, and AUC (49). Demonstration of improved outcomes, workflow benefit, or long-term impact is *not required* for clearance. In essence, clearance confirms that a model can perform a defined technical task under specified conditions, not that it will improve care across diverse real-world environments (50).

A defining feature of current FDA-cleared AI tools is that they are reviewed as *static models*. Although many originate from deep learning frameworks trained on large datasets, the specific version evaluated by regulators is fixed (from the time of clearance); parameters, preprocessing pipelines, and thresholds do not adapt post-clearance. Any modification that could affect performance typically triggers new validation requirements or additional submissions. This has clear implications for performance drift over time as hardware, acquisition conditions, or patient populations evolve.

Regulatory clearance is tightly coupled to the intended use statement, which specifies the population, input data type, and clinical task. Performance outside these bounds, for example, applying an ECG-based algorithm to different lead configurations, acquisition systems, or downstream clinical decisions is not evaluated during regulatory review and may substantially alter reliability. Reflecting this, the FDA has become increasingly stringent regarding the hardware systems on which AI algorithms are trained and validated, often requesting supporting data for each device platform on which the algorithm will be deployed.

For newer electrophysiology AI tools without a suitable predicate, many will likely enter through the De Novo pathway, which establishes the first special controls for a new device type and defines the boundaries within which clinical use is considered supported. In select cases, developers may also pursue Breakthrough Device designation when the tool addresses a meaningful unmet clinical need, accelerating FDA engagement even though the evidentiary requirements remain unchanged.

In parallel, the FDA has begun formalizing cardiovascular AI oversight through new AI-specific product classifications (for example, QXO for cardiovascular ML-based notifications, QXX for CAD, QYE for reduced ejection fraction, SAT for pulmonary hypertension, and SBQ for atrial fibrillation risk prediction). The emergence of these categories signals a more structured regulatory landscape, with clearer expectations for data requirements and intended use. For electrophysiologists, this means AI tools will increasingly arrive with sharper guardrails and more transparent boundaries around how they should be used.

Importantly, regulatory review does not address how AI outputs should be integrated into clinical decision-making, nor does it fully resolve how conflicts between algorithmic output and physician judgment should be managed, or how responsibility is assigned when AI-based recommendations influence care. These considerations extend beyond the scope of regulatory evaluation and necessarily fall to clinicians and post-market experience.

While this review emphasizes FDA pathways given their dominance in published AI-EP literature, electrophysiologists practicing outside the United States encounter different regulatory landscapes. The European Union's Medical Device Regulation governs CE marking, with requirements that historically emphasized technical documentation over prospective clinical validation, though recent revisions have increased scrutiny. In India, the Central Drugs Standard Control Organisation (CDSCO) regulates medical devices including AI-enabled systems, with many AI tools entering through import licensure or regulatory reliance on FDA or CE mark approval. For EPs, the practical implication is that regulatory clearance in one jurisdiction does not guarantee validation in the patient populations, healthcare systems, or device ecosystems where the tool will actually be deployed. Independent evaluation of performance in local practice settings remains essential regardless of regulatory status.

*For electrophysiologists, this creates a clear division of responsibility*: regulatory agencies act as gatekeepers for baseline safety and analytical performance within a defined use case, while clinicians remain responsible for determining whether an AI tool is appropriate for their patient population, data sources, and clinical risk context, and for recognizing when an algorithm is applied beyond the conditions under which it was validated.

**Fig. 3:**
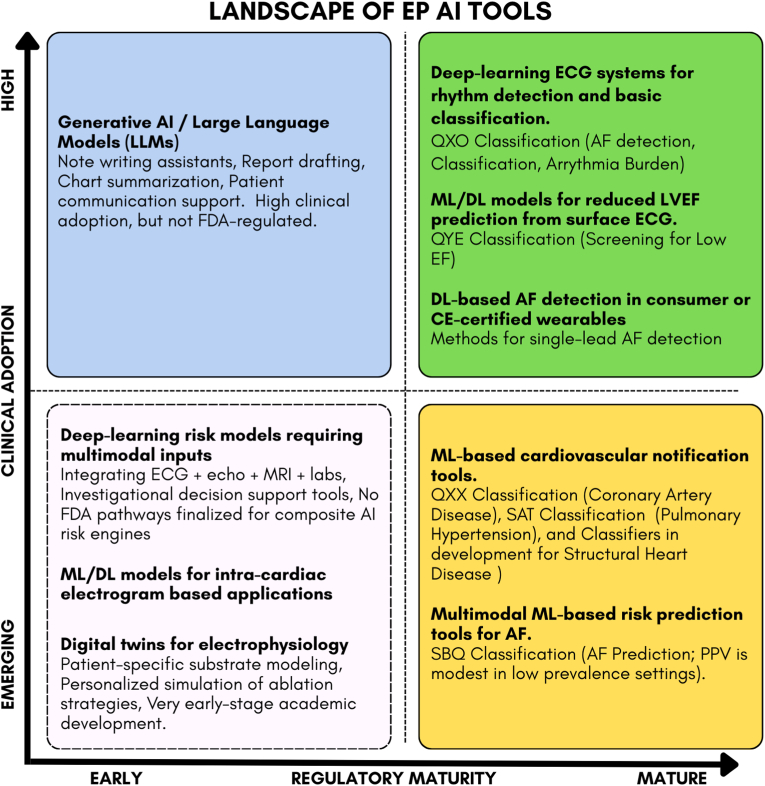
**Landscape of Electrophysiology AI Tools by Clinical Adoption and Regulatory Maturity.** This figure maps major categories of electrophysiology-relevant AI tools along two axes. The vertical axis reflects clinical adoption, ranging from *emerging* (early academic development, limited real-world use, no workflow integration) to *high adoption* (tools already used in practice, supported by publications, KOL acceptance, and reimbursement pathways). The horizontal axis reflects regulatory maturity, from *early* (no established FDA pathway or investigational use only) to *mature* (defined FDA product classifications or De Novo precedents). Acronyms: AF = atrial fibrillation; LVEF = left ventricular ejection fraction; ML = machine learning; DL = deep learning; LLM = large language model.

As FDA pathways and product classifications mature, Fig. 3 maps where EP AI tools currently sit along the regulatory and clinical adoption spectrum, highlighting areas where clinicians can confidently engage today and where their involvement will shape the standards that follow.

### 3.2 How to read and critically evaluate an AI study in electrophysiology

Electrophysiologists will increasingly encounter AI through journal publications, regulatory submissions, vendor claims, and institutional deployment decisions. While AI studies often report strong performance metrics, these results are frequently misinterpreted or overgeneralized. A structured approach to evaluating AI evidence is therefore essential to determine whether a given tool is appropriate for clinical use and under what conditions.

#### 3.2.1 Clinical problem definition

The first question is whether the AI system addresses a clinically meaningful problem. AI applications in EP may aim to classify rhythms, predict future risk, triage workflows, or assist documentation. These tasks differ fundamentally in clinical consequence. Tools that perform population level risk enrichment should not be interpreted as individual diagnostic tests. A common source of misapplication is assuming that high statistical performance equates to actionable clinical decision making without clarity on how outputs are intended to influence care.

#### 3.2.2 Data provenance and relevance

AI performance is inseparable from the data used to develop and validate the model. Studies should clearly describe data sources, including institutions, devices, lead configurations, acquisition settings, and patient demographics. Single system or single center datasets limit generalizability, particularly in electrophysiology where signal characteristics vary substantially across hardware, software preprocessing, and clinical environments. Ground truth definitions deserve particular scrutiny. Labels derived from expert consensus, downstream clinical decisions, or administrative codes introduce uncertainty that directly constrains clinical interpretation.

#### 3.2.3 Performance metrics in clinical context

Reported metrics must be interpreted relative to disease prevalence and intended use. Sensitivity, specificity, and AUC alone are insufficient to assess clinical utility. Positive predictive value is particularly critical in low prevalence conditions common in EP, where even small false positive rates can generate substantial downstream testing, cost, and patient anxiety. Internal validation almost always overestimates real world performance. External validation using independent datasets is a minimum requirement for clinical credibility.

#### 3.2.4 Generalizability and deployment conditions

Studies should demonstrate that performance extends beyond the development environment. Evaluation across institutions, devices, and time periods provides insight into robustness. Algorithms trained on narrow signal distributions may fail when exposed to routine variability in noise, lead placement, filtering, or patient behavior. Absence of such testing does not imply safety or reliability in practice.

#### 3.2.5 Failure modes and clinical oversight

AI systems should be evaluated not only by average performance but by how they fail. Clinically relevant questions include whether errors are predictable, whether uncertainty is communicated, and whether outputs can be verified or overridden. Tools intended for higher risk decisions demand stronger safeguards, such as human in the loop review or assistive rather than autonomous deployment. Lack of interpretability does not preclude clinical use, but it requires explicit acknowledgment of limitations and careful alignment with clinical risk.

#### 3.2.6 Regulatory clearance versus clinical readiness

Regulatory clearance establishes baseline analytical safety within a defined intended use. It does not demonstrate improved outcomes, workflow efficiency, or generalizability beyond validated conditions. Electrophysiologists must understand exactly what was reviewed, what assumptions were fixed, and what was not evaluated. Applying an AI tool outside its cleared population, data source, or task fundamentally alters its risk profile.

#### 3.2.7 Post deployment expectations

AI performance is not static in real world practice. Changes in devices, workflows, patient populations, or clinical behavior can degrade performance over time. Clinicians should expect the need for monitoring, revalidation, and recalibration. Responsibility for appropriate use remains with the physician, even when regulatory clearance has been granted.

Taken together, critical evaluation of AI studies requires moving beyond headline metrics to examine data integrity, validation rigor, failure behavior, and clinical integration. This framework enables electrophysiologists to distinguish promising tools from those that are technically impressive but clinically fragile.

**Fig. 4:**
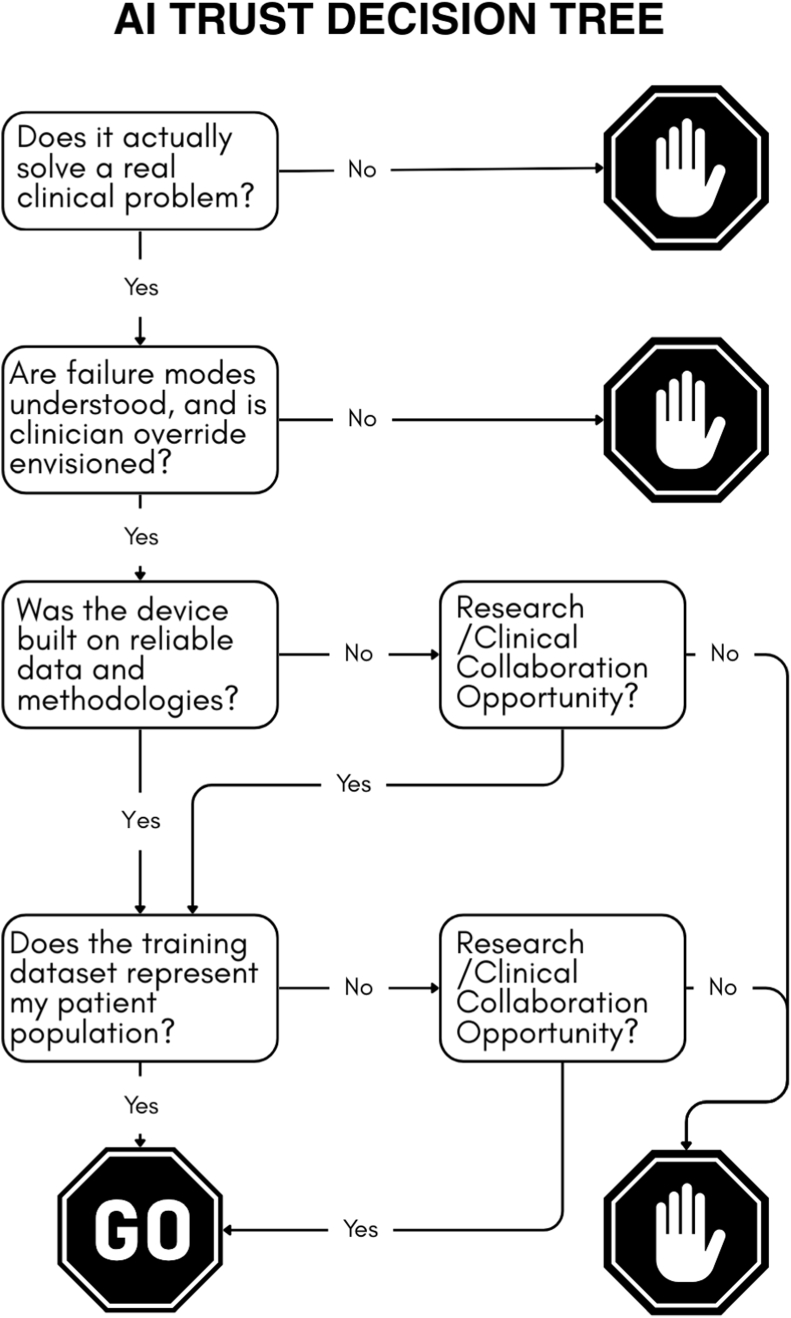
**Clinical Pearl: A practical decision tree to assess the trustworthiness of an AI tool**. A simple guide for electrophysiologists to evaluate an AI tool for clinical collaboration or adoption.

Fig. 4 provides a concise decision tree that electrophysiologists can use to assess the trustworthiness of an AI tool.

## 4 Shaping the next phase of AI in Electrophysiology

AI is entering a phase of rapid clinical adoption enabling signal interpretation, supporting earlier detection of disease states that exceed human perceptual limits, and reducing non-clinical burden through automation of documentation and data synthesis. In these domains, AI functions as an additional instrument, extending what electrophysiologists can see, process, and manage, rather than replacing clinical judgment.

A parallel frontier is emerging inside the EP lab itself. Machine-learning tools that analyze intracardiac electrograms to identify spatiotemporal activation patterns have now demonstrated clinically meaningful benefit in randomized trials and have received regulatory clearance, including superiority over conventional pulmonary vein isolation in a multicenter randomized controlled trial for persistent atrial fibrillation [5] and earlier multicenter studies showing standardized outcomes across operators and institutions [44]. These systems identify regions of spatiotemporal dispersion in real time during AF procedures, converting operator-dependent electrogram interpretation into an objective, reproducible process. Augmented reality platforms for electroanatomic map visualization have also received regulatory clearance, though clinical validation remains sparse [45]. Despite some regulatory maturity, intraprocedural AI guidance is still quite early from a clinical adoption standpoint with limited real-world implementation data [46]. The barriers are practical: EPs must determine whether these tools genuinely improve outcomes beyond pulmonary vein isolation, whether reimbursement pathways will sustain their use, and whether the added procedural workflow is justified by clinical benefit. The key distinction between this application and population-level screening tools is that intraprocedural AI directly influences therapeutic decisions in real time, raising the stakes for validation, interpretability, and failure mode understanding.

The more consequential opportunity lies ahead. Emerging approaches aim to move beyond population-level pattern recognition toward individualized modeling of cardiac electrophysiology. Digital twin frameworks [6,7], which integrate patient-specific anatomy, electrophysiology, imaging, and longitudinal data, represent a shift from probabilistic inference toward personalized simulation. Such models hold the potential to support treatment planning, predict response to therapy, and explore counterfactual clinical strategies in ways that static risk models cannot.

AI also offers a mechanism to extend expert-level support beyond traditional centers of excellence. Copilot and triage models can embed electrophysiology expertise into low-resource settings, supporting earlier identification of high-risk patients, prioritization of care, and escalation when needed. In health systems with constrained specialist access, including many regions in India, these applications may have greater practical impact than incremental performance gains in already well-resourced environments.

How this trajectory unfolds is not predetermined. The direction of AI development should be anchored in the real problems electrophysiologists face in daily practice, the data they judge to be clinically meaningful, and the standards they apply to validation and use. Tools built primarily because data are readily available or because a method aligns with prevailing technology trends risk solving the wrong problems, often with limited clinical utility or impact. This risk is particularly salient in emerging economies, where unmet needs are substantial and resources are constrained. AI guided by clinically grounded questions, physiologic plausibility, and real-world constraints has the potential to meaningfully reshape prevention, personalization, and access in electrophysiology.

## Declaration of competing interest

The authors declare the following financial interests/personal relationships which may be considered as potential competing interests:Charulatha Ramanathan reports a relationship with Carelog that includes: equity or stocks. Charulatha Ramanathan reports a relationship with Medtronic Inc that includes: consulting or advisory and travel reimbursement. Charulatha Ramanathan reports a relationship with Pulsheart that includes: consulting or advisory and equity or stocks. If there are other authors, they declare that they have no known competing financial interests or personal relationships that could have appeared to influence the work reported in this paper.
